# Supplementary material for: The causal relationship between the human gut microbiota and pyogenic arthritis: a Mendelian randomization study
Source: Front Cell Infect Microbiol. 2024 Nov 26;14:1452480. doi: 10.3389/fcimb.2024.1452480 (PMC11629706; doi:10.3389/fcimb.2024.1452480)
Supplement: Supplementary file 1 [file DataSheet1.zip › mendelian test/ebi-a-GCST90027828.csv_forest.pdf]

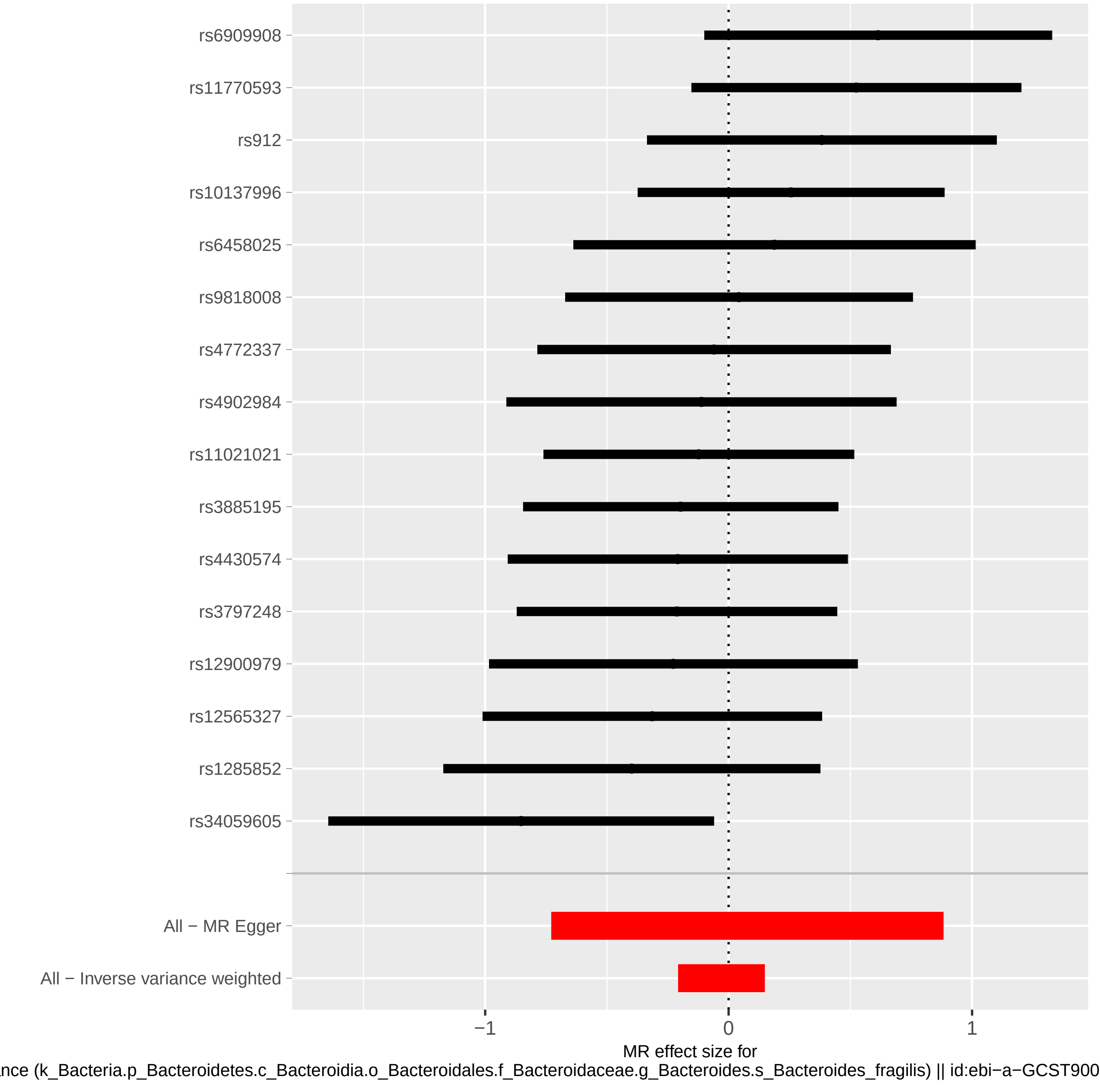
rs6909908
rs11770593
rs912
rs10137996
rs6458025
rs9818008
rs4772337
rs4902984
rs11021021
rs3885195
rs4430574
rs3797248
rs12900979
rs12565327
rs1285852
rs34059605
All − MR Egger
All − Inverse variance weighted
−1
0
1
MR effect size for
nce (k_Bacteria.p_Bacteroidetes.c_Bacteroidia.o_Bacteroidales.f_Bacteroidaceae.g_Bacteroides.s_Bacteroides_fragilis) || id:ebi−a−GCST900
